# Completion of the tuberculosis care cascade in a community‐based HIV linkage‐to‐care study in South Africa and Uganda

**DOI:** 10.1002/jia2.25065

**Published:** 2018-01-30

**Authors:** Adrienne E Shapiro, Alastair van Heerden, Torin T Schaafsma, James P Hughes, Jared M Baeten, Heidi van Rooyen, Elioda Tumwesigye, Connie L Celum, Ruanne V Barnabas

**Affiliations:** ^1^ Department of Global Health University of Washington Seattle WA USA; ^2^ Department of Medicine University of Washington Seattle WA USA; ^3^ Human Services Research Council Sweetwaters South Africa; ^4^ Department of Biostatistics University of Washington Seattle WA USA; ^5^ Vaccine and Infectious Diseases Division Fred Hutchinson Cancer Research Center Seattle WA USA; ^6^ Department of Epidemiology University of Washington Seattle WA USA; ^7^ School of Clinical Medicine Faculty of Health Sciences University of the Witwatersrand Johannesburg South Africa; ^8^ Integrated Community Based Initiatives Kabwohe Uganda

**Keywords:** tuberculosis, HIV, linkage, isoniazid preventive therapy

## Abstract

**Introduction:**

Tuberculosis (TB) is the leading cause of HIV‐associated mortality in Africa. As HIV testing, linkage to care and antiretroviral treatment initiation intensify to meet UNAIDS targets, it is not known what effect these efforts will have on TB detection and prevention. We aimed to characterize the TB care cascade of screening, diagnostic testing, treatment and provision of isoniazid preventive therapy (IPT) in a study of community‐based HIV screening and linkage to care and determine whether symptom screening results affected progress along the cascade.

**Methods:**

Between June 2013 and March 2015, HIV‐infected adults enrolled in the Linkages study, a multi‐site, community‐based, randomized HIV screening and linkage‐to‐care study in South Africa and Uganda. All participants were screened for TB symptoms at entry after testing positive for HIV and referred to local clinics for care. During the 9 month follow‐up, participants were periodically surveyed about clinic linkage and initiation of HIV care as well as subsequent TB testing, treatment, or IPT. We compared outcomes between persons with and without a positive symptom screen at baseline using descriptive statistics and Poisson regression to calculate relative risks of outcomes along the care cascade.

**Results and discussion:**

Of the 1,325 HIV‐infected adults enrolled, 26% reported at least one TB symptom at the time of HIV diagnosis. Loss of appetite and fever were the most commonly reported symptoms on a TB symptom screen. Despite 92% HIV linkage success, corresponding TB linkage was incomplete. Baseline TB symptoms were associated with an increased risk of a TB diagnosis (relative risk 3.23, 95% CI 1.51 to 6.91), but only 34% of symptomatic persons had sputum TB testing. Fifty‐five percent of participants diagnosed with TB started TB treatment. In South Africa, only 18% of asymptomatic participants initiated IPT after linkage to HIV care, and presence of symptoms was not associated with IPT initiation (relative risk 0.86 95% CI 0.6 to 1.23).

**Conclusions:**

HIV linkage to care interventions provide an opportunity to improve completion of the TB care cascade, but will require additional support to realize full benefits.

## 1 Introduction

Tuberculosis (TB) is a leading cause of mortality among persons with HIV/AIDS worldwide, causing nearly a third of HIV‐associated deaths 1. Early diagnosis and treatment of active TB and treatment of latent TB in HIV‐infected individuals reduce morbidity and mortality from TB. The WHO recommends intensive case‐finding for TB in people with HIV, consisting for adults and adolescents of a 4‐question symptom screen (presence of cough, fevers, night sweats and weight loss) and subsequent evaluation for TB if symptoms are present, as well as isoniazid preventive therapy (IPT) for HIV‐infected people without active TB 2.

**Figure 1:**
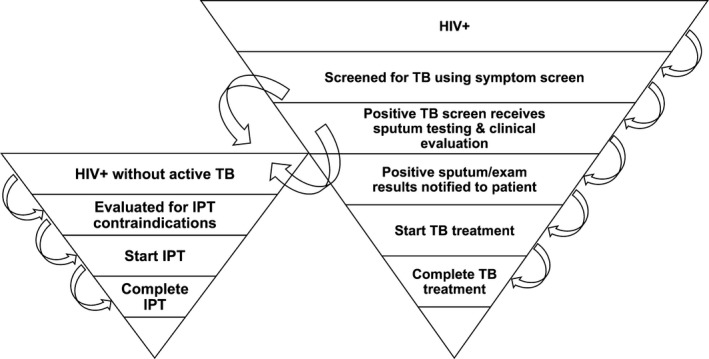
Schematic diagram of the TB care cascade. IPT= isoniazid preventive therapy.

Despite these recommendations, screening and linkage into care for TB treatment and IPT among HIV‐infected persons remains low 3. We evaluated participant progress through the stages of the “TB care cascade” (Figure 1), including screening yield, diagnostic testing, results of testing, and treatment or preventive therapy initiation in a community‐based HIV testing programme in South Africa and Uganda that was designed to increase knowledge of HIV serostatus and linkage to HIV care.

## 2 Methods

TB screening, testing and treatment were evaluated within a multisite, open‐labelled, randomized study (Linkages study) of community‐based HIV testing, counselling and linkage to HIV care conducted in two rural communities in KwaZulu‐Natal, South Africa and four in Sheema district, Uganda between 2013 and 2015, of which the methods and HIV outcomes have been described previously 4. Reported HIV prevalence in KwaZulu‐Natal was 28% 5 and there were 814 TB cases notified per 100,000 population in 2014 6. HIV prevalence was 10% in the district adjacent to Sheema 7 and TB prevalence was 159/100,000 nationally^1^. Briefly, HIV testing was performed at home or through mobile units. All participants found to be HIV‐infected were referred to local public clinics for HIV care, including initiation of ART if indicated. Four clinics in the Uganda site and six clinics in the South Africa site served as referral clinics for study participants. All referral clinics provided on‐site HIV counselling, testing, and ART initiation and could provide TB symptom screening and sputum collection for diagnostic testing, as well as TB treatment. X‐ray facilities were available at one clinic in Uganda and by referral to a hospital from the clinics in South Africa. HIV‐infected participants enrolled in the Linkages study were randomized to one of three arms for facilitation of linkage to care: counsellor reminder visits, counsellor accompaniment to clinic, and standard of care (no further assistance). Participants were also randomized to receiving a point‐of‐care CD4 count at enrolment or standard of care laboratory CD4 obtained at a clinic. At the enrolment visit, study staff administered a standardized 4‐question TB symptom screen to participants inquiring about cough, fever, night sweats and loss of appetite in the past 4 weeks. Results of symptom screening were provided to participants to take to clinic with their HIV test results and noted on the referral card. All subsequent investigations for TB were performed at the clinic per local clinical guidelines and practice.

Standard of care in South Africa for all HIV‐infected persons who reported at least one TB symptom was to obtain sputum‐based TB testing including a GeneXpert test and, if negative, a second sputum test for TB culture and drug susceptibility testing with possible chest X‐ray^8^. In Uganda, the standard of care for TB screening and diagnosis involved sputum microscopy and chest X‐ray, when available. GeneXpert was available in South Africa but not in the Uganda sites during the study period. Local guidelines recommended IPT for HIV‐infected persons without active TB in South Africa. IPT was recommended in Uganda but the drug was not available during the study period.

HIV‐positive participants were contacted periodically over 9 months after enrolment for follow‐up interviews. Depending on study arm, HIV‐positive participants received interim follow‐up visits at 1, 3 and 6 months, or no additional interim visits. All HIV‐positive participants were contacted at 9 months for an exit visit, survey, point‐of care CD4 count, and HIV viral load testing. At each follow‐up visit and the exit visit, all HIV‐positive participants were asked whether they (a) received sputum TB testing, (b) had a chest Xray, (c) received results from TB diagnostic tests, (d) received a diagnosis of TB, and (e) had started and completed either TB treatment or IPT during the follow‐up period. Results were verbal reports by participants. Study staff inspected medications available at home and participant clinic registration cards, but medications were not confirmed with local clinics.

Percentages were calculated for retention across each step of the TB “cascade” after excluding participants who had a known diagnosis of active TB at baseline as reported at the enrolment visit. Percentages of persons receiving a sputum test, receiving results of a sputum test, and initiating TB treatment were calculated separately for initially symptomatic and initially asymptomatic participants. Percentages initiating IPT at any point during the cascade were calculated for participants in South Africa only, since no IPT was available to Ugandan participants. Relative risks were determined using a Poisson regression model using robust variance estimation accounting for within‐household correlation, adjusted for age, sex, country, CD4 count and randomization arm.

Ethical approval to conduct the research was obtained from the institutional review boards of the University of Washington, the Human Sciences Research Council Research Ethics Committee, and the Ugandan National HIV/AIDS Research Committee. All study participants provided signed written informed consent or witnessed verbal informed consent with fingerprint signatures.

## 3 Results and discussion

Between June 2013 and February 2015, 15,332 persons received HIV testing and counselling, of whom 1,325 (8.6%) tested positive for HIV and were enrolled in the Linkages Study (Table 1).

**Table 1:** Demographic and clinical characteristics of participants, by results of TB symptom screen at enrolment

	No TB Symptoms at Baseline N = 976 (74%)	Any TB Symptoms at Baseline N = 349 (26%)	Total N = 1325
Country site			
South Africa	698 (72)	222 (64)	920 (69)
Uganda	278 (28)	127 (36)	405 (31)
Sex			
Male	256 (26)	123 (35)	379 (28)
Female	720 (74)	226 (65)	946 (71)
Age			
16 to 24	201 (21)	66 (19)	267 (20)
25 to 34	432 (44)	128 (37)	560 (42)
>34	343 (35)	155 (44)	498 (38)
Median (IQR) CD4 (N = 1019)	510 (377 to 676) (N = 757)	441 (302 to 621) (N = 262)	500 (355 to 654)
Taking TB treatment at enrolment	3 (0.3)	7 (2)	10 (0.75)
Median number TB Symptoms (IQR)	0	2 (1 to 2)	0 (0 to 1)
TB Symptoms reported			
Cough	–	135 (39)	
Fever	–	169 (48)	
Night Sweats	–	153 (44)	
Loss of appetite	–	190 (54)	
Visited HIV care facility (linkage by month 9)	894 (92)	324 (93)	1218 (92)
Diagnosis of TB			
South Africa	11 (2)	13 (6)	24 (3)
Uganda	0 (0)	2 (2)	2 (1)

There were 405 participants from Uganda and 920 from South Africa. Baseline CD4 count was available for 1019 participants; median baseline CD4 was (500 cells/μl, IQR 355‐654). CD4 counts were missing from some participants who were randomized to receive clinic‐based CD4 only and whose results were not available on clinic registration cards. Treatment for active TB was reported by less than one percent (10/1325) of participants at the enrolment visit. At enrolment, 973 (74%) participants reported no symptoms of active TB and 154 (11%), 106 (8%), 64 (5%) and 18 (1%) reported 1, 2, 3 and 4 symptoms of TB in the prior 4 weeks respectively. Among participants, loss of appetite was most common 185/1325 (14%), followed by fever (166/1325, 13%), night sweats (149/1325, 11%) and cough (130/1325, 10%). Linkage to HIV clinics was high (92%) and there was no difference in clinic linkage among patients with or without TB symptoms at baseline (RR=1.01, 95% CI 0.98 to 1.05, *p* = 0.38).

**Figure 2:**
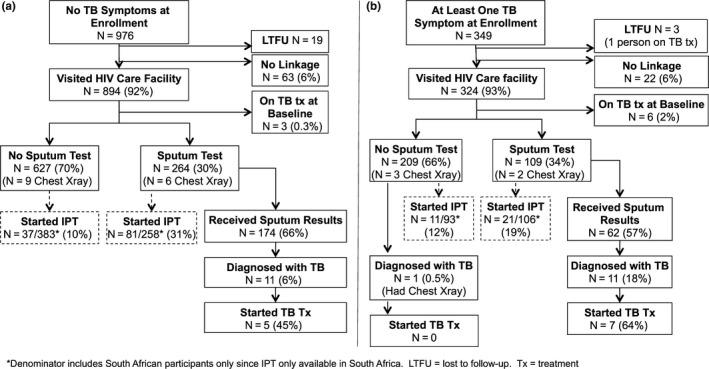
Flow diagrams showing participant progress through TB care cascade during 9‐month study follow‐up. Denominators include all participants in prior box unless specified otherwise. (a) No baseline symptoms (b) One or more baseline symptoms.

After 9 months of HIV follow‐up, all but 22 participants had follow‐up data available. Of the 109/318 (34%) persons with ≥1 TB symptom and who visited an HIV care facility reported having sputum collected for TB testing, (Figure 2). Among participants not already on TB treatment reporting symptoms at enrolment, 12/340 (4%) were diagnosed with active TB, compared to 11/954 (1%) of participants reporting no baseline symptoms (RR = 3.23, 95% CI 1.51 to 6.91, *p* = 0.002).

Only 55% (12/22, 95%CI 32‐76%) of HIV‐infected persons diagnosed with active TB initiated TB treatment. Ten percent (32/324) of participants who reported symptoms and 13% (119/891) of persons without TB symptoms initiated IPT after attending clinic. There was no difference in IPT initiation in the follow‐up period between persons with and without TB symptoms at baseline (RR = 0.86, 95% CI 0.6 to 1.23, *p* = 0.4). Among participants who were symptomatic at baseline, 34% (11/32) who started IPT denied any sputum or chest X‐ray testing before starting IPT.

In a community‐based programme that achieved over 90% uptake of HIV testing and linkage to HIV care in South Africa and Uganda, all HIV‐infected participants were screened for TB with a symptom screen at baseline. Clinic‐initiated follow‐up of screening for TB based on symptoms was incomplete, uptake of IPT was low, and initiation of TB treatment occurred in only half of participants diagnosed with TB despite a high rate of clinic attendance, linkage into HIV care, and contact with health care providers. In this population, overall 37% of participants and 66% of those meeting eligibility criteria at the time (CD4 < 350, pregnant, or with active TB) initiated ART during the study, and 85% were virologically suppressed at month 9 of follow‐up 4.

The 90‐90‐90 targets set by UNAIDS promote ambitious targets for screening for HIV (90% of HIV‐infected people knowing their status), linking HIV‐infected people into care (90% of people testing positive linking to care), and achieving virological suppression with antiretroviral therapy (90% of people linked to care with virological suppression) 9. The Linkage Study exceeded the 90% HIV linkage goal and identified over a quarter of HIV‐infected participants with at least one symptom on a TB symptom screen. Despite high linkage to HIV care and highly prevalent positive symptom screens, subsequent attrition along the steps of the TB cascade exceeded the drop‐off along the progression of the HIV care continuum seen in this setting. Only a third of study participants ultimately had clinic‐based follow‐up of positive TB symptom screens with sputum testing, less than 15% received IPT, and less than 60% of people diagnosed with TB received treatment for TB. The observed 2% prevalence of TB based on reported diagnoses was lower than predicted for this population compared to similar populations of ART‐naïve, HIV‐infected persons investigated for TB systematically with more sensitive diagnostic tools, suggesting under‐diagnosis in this setting 10, 11. TB treatment was not begun in nearly half of participants who did receive a diagnosis of TB—both of these indicators identify significant drop‐offs in the cascade from TB symptom screening to treatment. IPT uptake even where available was low, with ten percent of all participants who were asymptomatic at baseline receiving IPT and 18% of asymptomatic participants in South Africa (where IPT was available in clinics) receiving IPT. In addition, 33% of persons with baseline symptoms who received IPT reported no further investigations for active TB, raising concern for promotion of isoniazid resistance if IPT is provided to persons with undiagnosed active TB. Some asymptomatic participants received a sputum test, although it was not possible to determine whether this was done in response to new symptoms arising after the baseline screen. Interestingly, more participants who had no symptoms at baseline and received a sputum test went on to start IPT than participants who did not have a sputum test, who were presumptively eligible for IPT. These findings may represent inconsistent practices across clinics, or may reflect variable provider awareness and priorities, with some providers more inclined to provide IPT after additional reassurance that TB had been excluded, while others provide IPT without adequate exclusion of active TB.

These TB screening outcomes are consistent with other clinic‐based studies that have reported progressive drop‐off in the TB care cascade in Ugandan and South African clinics 12, 13. However, this study differs in that it evaluated participants attending multiple clinics in two countries in conjunction with a community‐based HIV testing and linkage programme that increased the number of HIV‐infected persons referred to nearby clinics and performed the initial TB symptom screening prior to clinic linkage. The low proportion who received sputum testing, including those who went on to receive IPT, indicates a need to focus on improving clinics’ use of symptoms to triage to sputum collection for GeneXpert and/or culture and chest X‐ray to rule out active TB. Importantly, the findings here show that the gaps along the TB care cascade persist in the setting of an otherwise effective HIV linkage programme.

The study had several limitations. TB testing and treatment initiation were all reported by participants and were not independently confirmed with clinic or laboratory records. Self‐reported outcomes may have decreased reliability, or be subject to recall bias. However, the duration and intensity of both TB treatment and IPT increases the likelihood of patient recall accuracy. The total number of TB diagnoses was low, limiting precision in estimates of treatment uptake. We were also unable to ascertain reasons at the clinic level for drop‐off at each stage of the cascade.

Despite these limitations, this study provides important insights into operational TB challenges in an otherwise successful HIV linkage programme, with significant room for improvement in HIV‐linked IPT provision. TB is the leading cause of HIV‐associated mortality in sub‐Saharan Africa, and TB preventive therapy in addition to ART is projected to be essential to reducing TB incidence and deaths 14. The TEMPRANO study demonstrated that IPT in combination with ART significantly reduced the risk of mortality and severe AIDS‐related illness in HIV‐infected persons 15. IPT was safe and effective, with no additional adverse events or additional drug resistance observed during IPT. Barriers to providing IPT include lack of availability, provider concern about promoting resistance, lack of clarity about appropriate candidates, and viewing IPT as a low priority among competing healthcare needs 16, 17. The variability observed in this study related to TB symptom investigation and IPT provision highlight a need to reinforce clear, consistent policies to ensure TB symptoms are investigated, but that IPT is provided to patients when active TB is ruled out.

Scale‐up of HIV linkage represents a potential opportunity to identify and treat TB cases and prevent TB with IPT, but as shown in the Linkages Study, HIV linkage alone is not sufficient for this to occur without additional support for implementation of TB services and HIV treatment. The WHO changed its recommendations in 2015 to remove CD4 thresholds for ART initiation and many countries have updated their national policies accordingly, including South Africa and Uganda. While this important change may decrease the gap between people identified with HIV and people receiving ART, it is not clear what effect it will have on the TB care cascade. The optimal approach to integration of TB screening and linkage to TB care into HIV linkage efforts needs to be determined, because increased access to rapid diagnostic tools and fast‐tracked empiric TB therapy have not decreased mortality 18, 19.

## Conclusions

Interventions to address gaps in the TB care cascade, promote linkage to care after TB screening, and increase adoption of IPT need to be implemented in HIV linkage and treatment programmes. As HIV linkage programmes are strengthened to identify more HIV‐positive people and connect them with HIV care, it is essential that TB services be concomitantly strengthened in order to yield the maximum mortality benefit to patients from these programmes.

## Competing interests

The authors declare that they have no competing interests.

## Authors’ contributions

AES contributed to the analysis and interpretation of data. AvH contributed to the concept and design, data acquisition, and interpretation. TTS contributed to the data analysis and interpretation. JPH contributed to the study design, analysis, and interpretation. HvR contributed to the concept and design, data acquisition, and interpretation. ET contributed to the concept and design and data acquisition. JMB contributed to the study conception and interpretation of data. CC contributed to the study conception, acquisition and interpretation of data. RVB contributed to the study conception, data acquisition and interpretation. All authors have been involved in drafting the manuscript or revising it critically for intellectual content and have given approval for the final version to be published.
